# Supplementary material for: Prioritization of candidate genes for a South African family with Parkinson’s disease using in-silico tools
Source: PLoS One. 2021 Mar 26;16(3):e0249324. doi: 10.1371/journal.pone.0249324 (PMC7997022; doi:10.1371/journal.pone.0249324)
Supplement: S1 Table — (PDF) [file pone.0249324.s001.pdf]

S1 Table. Clinical and demographic information on members of a South African family with Parkinson's disease

	**Affected**					**Unaffected**			
**Individual ID**	III-8	III-7	IV-2	III-6	III-2	III-4	IV-1	III-1	III-5
**Gender**	Male	Male	Male	Female	Female	Male	Male	Female	Male
**Age in 2021 (years)**	66	72	47	75	83	72	46	74	76
**Age at evaluation**	55	60	44	72	77	62	35	68	67
**Age at onset (years)**	48	42	37	70	75	N/A	N/A	N/A	N/A
**Childhood symptoms**	Reported to have walked on toes since childhood	None	None	None	None	N/A	N/A	N/A	N/A
**Initial symptoms**	Muscle spasm in left arm, poor balance, walking on toes	Impaired concentration Weakness of legs, and difficulty walking	Difficulty with walking and balance	Cramping lower legs	Difficulty with fine motor ADL; left hand tremor	N/A	N/A	N/A	N/A
**Olfaction**	Impaired	Normal	Always poor	Normal	Impaired	N/A	N/A	N/A	N/A
**Excessive sweating**	No	Yes	No	No	No	N/A	N/A	N/A	N/A

**RBD**	No	No	No	No	Yes	N/A	N/A	N/A	N/A
**Orthostatic hypotension**	Yes	Onset age 45	No	No	No	N/A	N/A	N/A	N/A
**Bladder function**	Mild difficulty	Normal	Normal	Normal	Normal	N/A	N/A	N/A	N/A
**Constipation**	Yes	None	No	No	Yes	N/A	N/A	N/A	N/A
**Sleep impairment**	No	Yes	No	No	Yes	N/A	N/A	N/A	N/A
**Dystonia**	Initially walked on toes. Dystonia of left leg after medication	Dystonic flexion of left arm when walking	Facial	No	No	N/A	N/A	N/A	N/A
**Tremor**	Developed later, predominantly action tremor, with intention component	Action/intention	No	No	Initial complaint	N/A	N/A	N/A	N/A
**Bradykinesia**	Marked, asymmetrical, predominantly upper limbs	Markedly asymmetrical in upper and lower limbs	Mild bilateral	Mild bilateral	Mild bilateral	N/A	N/A	N/A	N/A
**Rigidity**	Bilateral cogwheel	Marked asymmetry	Mild bilateral, asymmetrical	Normal tone	Unilateral	N/A	N/A	N/A	N/A
**Gait**	Shuffling gait, with absent arm swing	Broad-based, diminished arm swing	Mild slowing	Normal	Slowed	N/A	N/A	N/A	N/A
**Additional findings**	Diminished vibration sense in toes	Diminished vibration and loss of joint position sense in toes. No	Micrographia	None	None	N/A	N/A	N/A	N/A

		evident axonal or demyelinating changes in tibial, peroneal or sural conductions.							
**Total daily dose levodopa (mg)**	1000	600	0	0	0	N/A	N/A	N/A	N/A
**Levodopa responsive**	Yes	Yes	N/A	N/A	Not known	N/A	N/A	N/A	N/A
**Blood pressure**	No orthostatic drop	No orthostatic drop	No orthostatic drop	No orthostatic drop	No orthostatic drop	N/A	N/A	N/A	N/A
**Reflexes**	Hyperreflexia present	Hyperreflexia in lower limbs	Hyperreflexia in lower limbs, right > left	Normal	Normal	N/A	N/A	N/A	N/A
**Brain CT scan**	No abnormality except for internal capsule lacune	Not done	Not done	Not done	Not done	N/A	N/A	N/A	N/A
**UPDRS**	41	37	16	11	15	N/A	N/A	N/A	N/A
**MOCA**	26	27	30	28	Not done	N/A	N/A	N/A	N/A

ADL, Activities of Daily Living; RBD, REM sleep behavior disorder; MOCA, Montreal Cognitive Assessment; N/A, not applicable; UPDRS, Unified Parkinson Disease Rating Scale
