# Supplementary material for: Prioritization of candidate genes for a South African family with Parkinson’s disease using in-silico tools
Source: PLoS One. 2021 Mar 26;16(3):e0249324. doi: 10.1371/journal.pone.0249324 (PMC7997022; doi:10.1371/journal.pone.0249324)
Supplement: S2 Table — (PDF) [file pone.0249324.s002.pdf]

S2 Table. Summary of whole exome sequencing (WES) metrics in the three affected individuals

	**Individual III-7**	**III-8**	**IV-2**
Total number of reads	136,048,746	127,298,302	114,750,902
Non-duplicated reads	90,255,052	97,195,484	85,612,771
Reads aligned to target	82,045,320 (90,9%)	83,570,035 (86%)	74,665,623 (87,2%)
Mean target coverage (%)	88.12	79.88	71.04
Total number of variants	22,318	22,566	22,531
