# Supplementary material for: Prioritization of candidate genes for a South African family with Parkinson’s disease using in-silico tools
Source: PLoS One. 2021 Mar 26;16(3):e0249324. doi: 10.1371/journal.pone.0249324 (PMC7997022; doi:10.1371/journal.pone.0249324)
Supplement: S1 Fig — (PDF) [file pone.0249324.s004.Pdf]

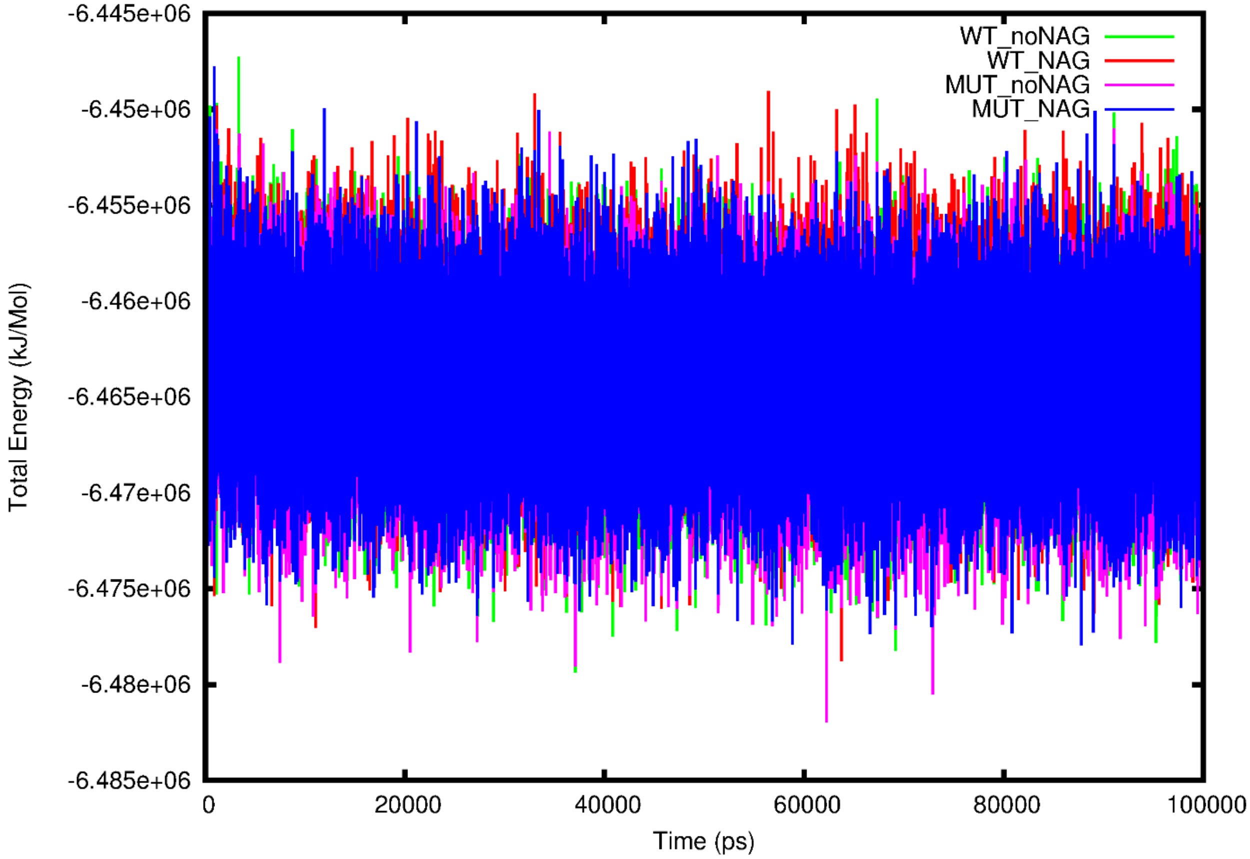

S1 Fig. Total energy of the four systems of NRXN2 (WT_noNAG, MUT_noNAG, WT_NAG and MUT_NAG) over 100 ns.

Abbreviations: WT, wild-type; MUT, p.G849D mutant; NAG, N-acetyl-D-glucosamine
