# Supplementary figures and images for: Prioritization of candidate genes for a South African family with Parkinson’s disease using in-silico tools
Source: PLoS One. 2021 Mar 26;16(3):e0249324. doi: 10.1371/journal.pone.0249324 (PMC7997022; doi:10.1371/journal.pone.0249324)

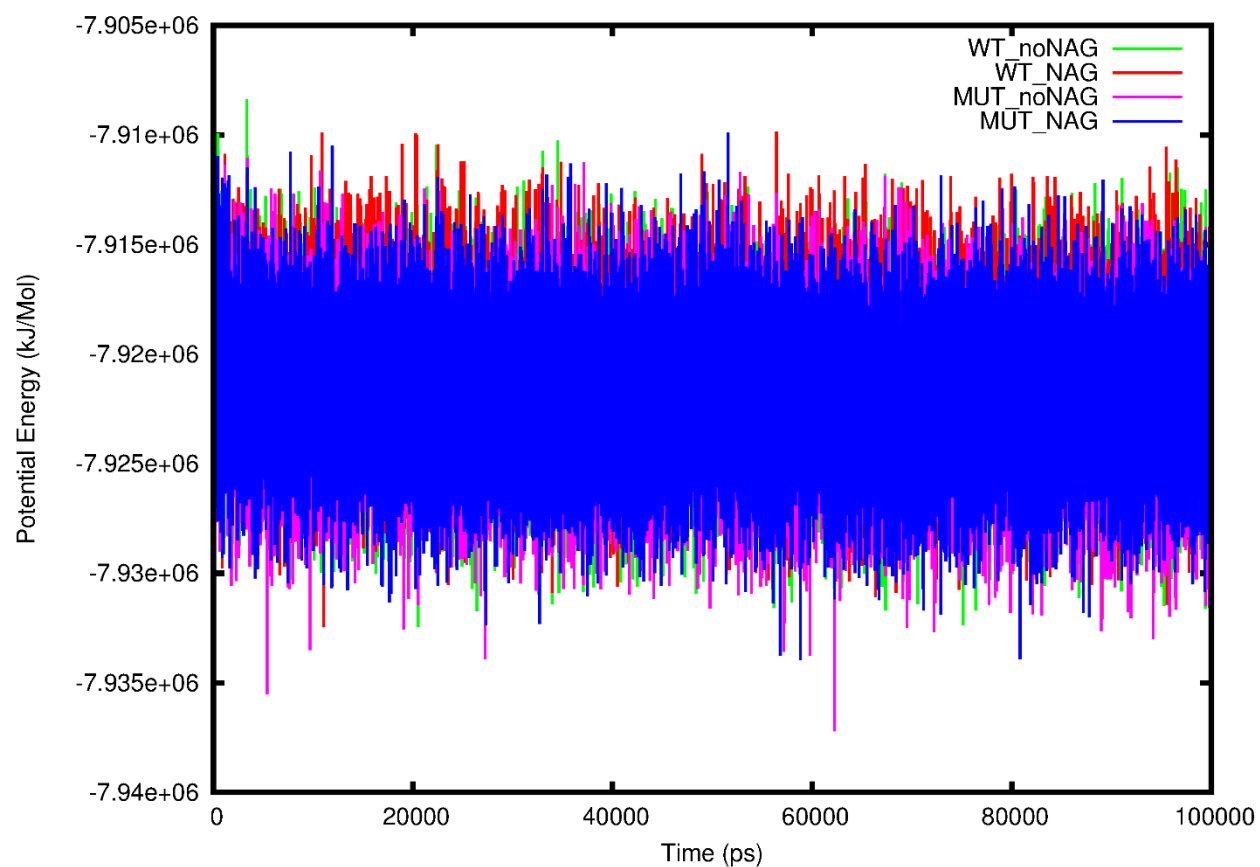

**S2 Fig.** Potential energy of the four systems of NRXN2 (WT\_noNAG, MUT\_noNAG, WT\_NAG and MUT\_NAG) over 100 ns.

Supplement: S2 Fig — (PDF) [file pone.0249324.s005.pdf]
