# Supplementary material for: Prioritization of candidate genes for a South African family with Parkinson’s disease using in-silico tools
Source: PLoS One. 2021 Mar 26;16(3):e0249324. doi: 10.1371/journal.pone.0249324 (PMC7997022; doi:10.1371/journal.pone.0249324)
Supplement: S4 Fig — Figure generated using PoseView. Dashed lines show hydrogen bond contacts formed NAG and NRNX2 residues. (PDF) [file pone.0249324.s007.pdf]

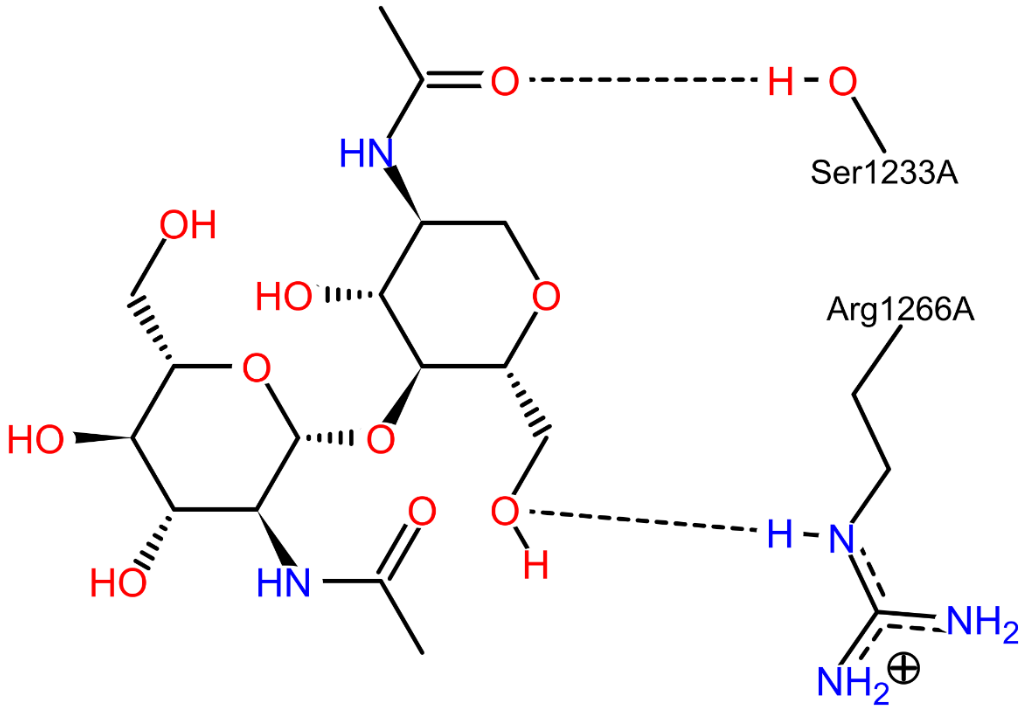

S4 Fig. 2D Interaction diagram showing polar contacts formed between NRXN2 residues and sugar moiety NAG. Figure generated using PoseView. Dashed lines show hydrogen bond contacts formed NAG and NRNX2 residues.
