# Supplementary material for: Prioritization of candidate genes for a South African family with Parkinson’s disease using in-silico tools
Source: PLoS One. 2021 Mar 26;16(3):e0249324. doi: 10.1371/journal.pone.0249324 (PMC7997022; doi:10.1371/journal.pone.0249324)
Supplement: S8 Fig — MEAN + STDEV (1.71mm ± 0.52, 2.26mm ± 0.52, 1.27mm ± 0.46 and 2.14mm ± 0.79). Line colours: WT_noNAG = green, MUT_noNAG = light magenta, WT_NAG = red and MUT_NAG = blue. (PDF) [file pone.0249324.s011.pdf]

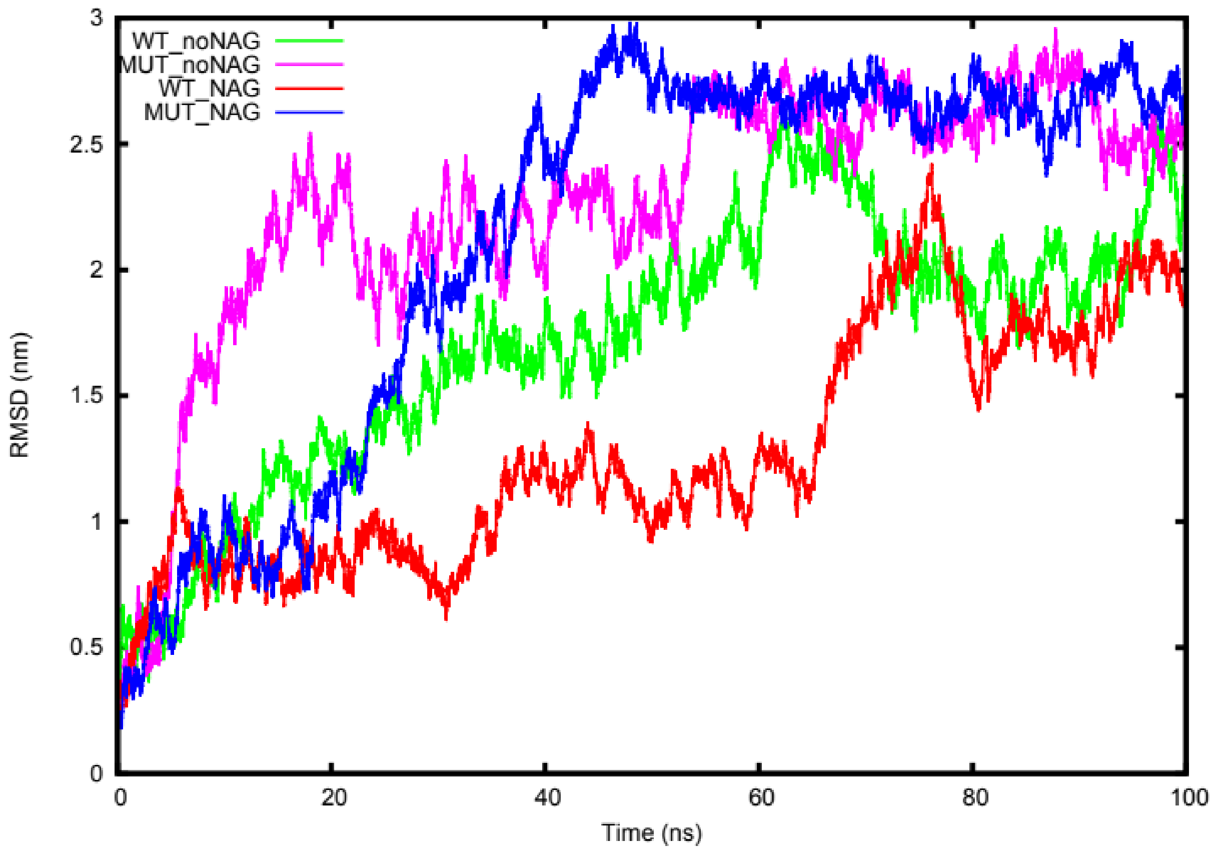

S8 Fig. RMSD deviation of the backbone atoms for the four systems of repeat 1 NRXN2. MEAN + STDEV (1.71mm ± 0.52, 2.26mm ± 0.52, 1.27mm ± 0.46 and 2.14mm ± 0.79). Line colors: WT_noNAG = green, MUT_noNAG = light magenta, WT_NAG = red and MUT_NAG = blue.
