# Supplementary material for: Prioritization of candidate genes for a South African family with Parkinson’s disease using in-silico tools
Source: PLoS One. 2021 Mar 26;16(3):e0249324. doi: 10.1371/journal.pone.0249324 (PMC7997022; doi:10.1371/journal.pone.0249324)
Supplement: S9 Fig — (PDF) [file pone.0249324.s012.pdf]

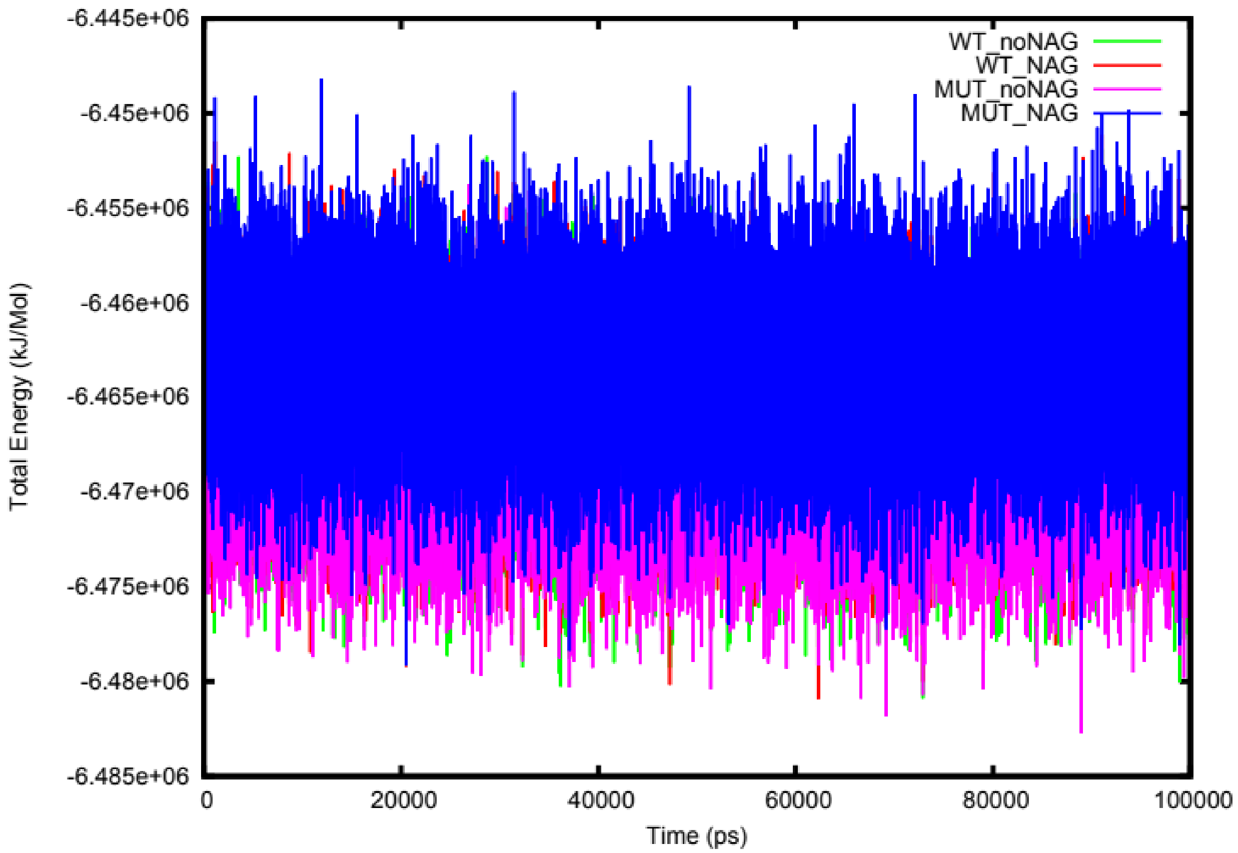


**S9 Fig.** Total energy of the four systems of the repeat 2 of NRNX2 (WT_noNAG, MUT_noNAG, WT_NAG and MUT_NAG) over 100 ns.
