# Supplementary material for: Prioritization of candidate genes for a South African family with Parkinson’s disease using in-silico tools
Source: PLoS One. 2021 Mar 26;16(3):e0249324. doi: 10.1371/journal.pone.0249324 (PMC7997022; doi:10.1371/journal.pone.0249324)
Supplement: S12 Fig — Line colours: WT_noNAG = green, MUT_noNAG = light magenta, WT_NAG = red and MUT_NAG = blue. (PDF) [file pone.0249324.s015.pdf]

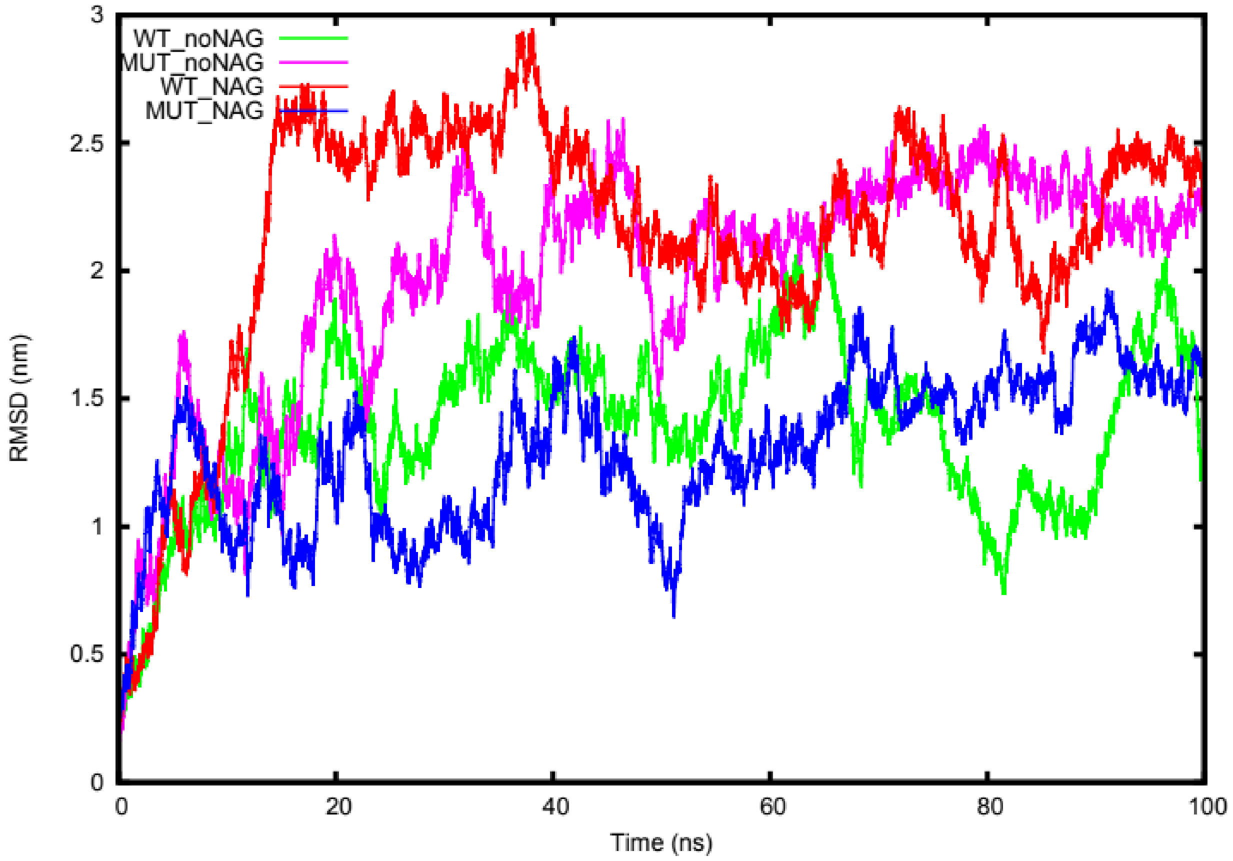

S12 Fig. RMSD deviation of the backbone atoms for the four systems of repeat 2 NRXN2, (1.40mm ± 0.33, 1.98mm ± 0.45, 2.15mm ± 0.51 and 1.29mm ± 0.29). Line colors: WT_noNAG = green, MUT_noNAG = light magenta, WT_NAG = red and MUT_NAG = blue.
